# Detection of selective sweeps in cattle using genome-wide SNP data

**DOI:** 10.1186/1471-2164-14-382

**Published:** 2013-06-07

**Authors:** Holly R Ramey, Jared E Decker, Stephanie D McKay, Megan M Rolf, Robert D Schnabel, Jeremy F Taylor

**Affiliations:** 1 Division of Animal Sciences, University of Missouri, Columbia, MO 65211, USA; 2 Department of Animal Science, University of Vermont, Burlington, VT 05405, USA; 3 Department of Animal Science, Oklahoma State University, Stillwater, OK 74074, USA; 4 S135B Animal Sciences, University of Missouri, 920 East Campus Drive, Columbia, MO 65211-5300, USA

**Keywords:** Selective sweep, Reduced heterozygosity, Signatures of selection, Single nucleotide polymorphisms

## Abstract

**Background:**

The domestication and subsequent selection by humans to create breeds and biological types of cattle undoubtedly altered the patterning of variation within their genomes. Strong selection to fix advantageous large-effect mutations underlying domesticability, breed characteristics or productivity created selective sweeps in which variation was lost in the chromosomal region flanking the selected allele. Selective sweeps have now been identified in the genomes of many animal species including humans, dogs, horses, and chickens. Here, we attempt to identify and characterise regions of the bovine genome that have been subjected to selective sweeps.

**Results:**

Two datasets were used for the discovery and validation of selective sweeps via the fixation of alleles at a series of contiguous SNP loci. BovineSNP50 data were used to identify 28 putative sweep regions among 14 diverse cattle breeds. Affymetrix BOS 1 prescreening assay data for five breeds were used to identify 85 regions and validate 5 regions identified using the BovineSNP50 data. Many genes are located within these regions and the lack of sequence data for the analysed breeds precludes the nomination of selected genes or variants and limits the prediction of the selected phenotypes. However, phenotypes that we predict to have historically been under strong selection include horned-polled, coat colour, stature, ear morphology, and behaviour.

**Conclusions:**

The bias towards common SNPs in the design of the BovineSNP50 assay led to the identification of recent selective sweeps associated with breed formation and common to only a small number of breeds rather than ancient events associated with domestication which could potentially be common to all European taurines. The limited SNP density, or marker resolution, of the BovineSNP50 assay significantly impacted the rate of false discovery of selective sweeps, however, we found sweeps in common between breeds which were confirmed using an ultra-high-density assay scored in a small number of animals from a subset of the breeds. No sweep regions were shared between indicine and taurine breeds reflecting their divergent selection histories and the very different environmental habitats to which these sub-species have adapted.

## Background

The transition from hunter-gather lifestyles to permanent dwelling societies was facilitated by both plant and animal domestication [1]. The domestication of cattle occurred between 8,000 and 10,000 years ago and led to changes in the genome of the species due to the effects of demography and selection [2,3]. Much of the variation within the genetically diverse ancestral population was either lost due to the limited sampling of animals within the sites of domestication or was partitioned into the subpopulations which went on to become recognised as distinct breeds. Selection for the phenotypes contributing to domesticability, biological type (draught, milk, meat) and the aesthetically appealing morphologies that have become breed hallmarks (polled, coat colour and patterning [4-8]) have also impacted the extent and distribution of variability within the genome.

Strong on-going selection for variants of large effect leads to a loss of variation within the chromosomal region flanking the selected variant and ultimately the complete fixation of the haplotype which harbours the variant. This phenomenon is known as the “hitch-hiking effect” [9] and a region of the genome in which artificial selection has driven a haplotype to complete fixation is defined as having been subjected to a “selective sweep”. Such regions may also occur within the genome due to random drift and these regions are not distinguishable from regions subjected to selective sweeps. Selective sweep studies differ from the classical “forward” genetics approach, which progresses from a phenotype to the identification of underlying causal genes and mutations. Rather, they follow a “reverse” genetics approach that begins with a signature of selection and attempts to infer the selected mutation and its associated phenotype [1]. An important reason for seeking selective sweeps is that these regions can elucidate the identities of genes and mutations with large phenotypic effect even if they are no longer segregating within any one population and thus cannot be detected by forward genetics without the formation of expensive crosses.

Several methods have been used to identify regions of the genome which have been subjected to selective sweeps, including those based on modeling allele frequency spectra, linkage disequilibrium and haplotype structure [10-12]. These approaches require the use of high-density single nucleotide polymorphism (SNP) data which have previously been shown to be useful for detecting selective sweeps in human populations [13,14]. Studies aimed at localizing signatures of selection and selective sweeps have been performed in many animal species using SNP and microsatellite loci. These studies have pointed to interesting phenotypes which are important to understanding the nature of historic natural and artificial selection applied to these species. In chicken, selective sweeps have been found to involve loci believed to be inherent to domestication and include *BCDO2* which controls yellow and white skin colours, *SEMA3A* which plays a role in axonal path-finding important in brain development, and *THSR* which is postulated to derestrict the regulation of seasonal reproduction [15]. Selective sweeps have been found in the dog genome at *TRYP1* which controls black coat colour in Large Munsterlanders and at *FGFR3* in Dachshunds [16]. *FGFR3* mutations cause achondroplasia in humans and cattle. Other studies in dogs have identified a sweep surrounding *IGF1* which is responsible for size variation [17] and in a genomic region for which the selected phenotype is unknown in Boxers [18]. These sweeps range in size from 28 kb to 40 Mb suggesting considerable variation in the intensity of selection and also in the population census sizes of these breeds. A ~75 kb selective sweep at a locus influencing stature in the horse is upstream of a transcription factor (*LCORL*) that is associated with variation in human height [19]. A 28 kb selective sweep in a region of the swine genome harbouring *IGF2* has been implicated with selection for increased muscle mass and decreased fat deposition [20]. More recently, whole genome resequencing has been utilized in swine to identify selective sweeps in the *NR6A1*, *PLAG1* and *LCORL* genes which are associated with an increased number of vertebrae and an elongation of the animal’s back [21].

Domestic animals have been demonstrated to be excellent models for genetic studies due to the availability of extensive pedigrees and because, as species, they are frequently more genetically diverse than human [22]. However, relatively few large-scale selective sweep studies have been conducted in cattle to elucidate the genes which have historically been selected by humans to create the existing diversity of breeds and specialised biological types for draught, milk and meat production [23]. Rather, studies to date have tended to focus on specific breeds, individual biological type, or chromosomal regions [24-28]. In this study, we sought to identify signatures of completed selective sweeps genome-wide using 6,373 animals from 14 breeds genotyped with the high-density BovineSNP50 assay and 58 individuals from five breeds genotyped with the ultra-high-density Affymetrix BOS 1 prescreening assay (AFFXB1P). The sampled animals represent 14 meat and milk producing breeds as well as the taurine and indicine sub-species (Table 1). The detected sweeps were considered to be validated if they were found in more than one breed or if they were found in analyses of both the BovineSNP50 and AFFXB1P data. Our goals are to ultimately identify the selected mutations and phenotypes subjected to selection by our ancestral herdsmen in the processes of domestication and formation of breeds and biological types. We present here a comprehensive genome-wide analysis of selective sweeps in cattle.

**Table 1:** Summary for genotyped individuals

**Breed**^**1**^	**Origin**	**Primary historical use**	**Contiguous BovineSNP50 loci**^**2**^	**Number monomorphic SNP50 loci**	**Number BovineSNP50 individuals**	**Number AFFXB1P individuals**
Angus	Scotland	Beef	6	8,443	2,918	23
Braunvieh	Switzerland	Beef	5	7,405	142	
Charolais	France	Beef	5	5,884	44	
Hanwoo	Korea	Beef, Draught	5	8,353	48	11
Hereford	UK	Beef	6	8,154	812	
Limousin	France	Beef	6	8,430	261	
Salers	France	Beef	6	8,409	72	
Shorthorn	UK	Beef, Dairy	6	8,558	108	
Simmental	Switzerland	Beef, Dairy	6	6,599	123	6
Brown Swiss	Switzerland	Dairy	9	12,553	74	
Finnish Ayrshire	Scotland	Dairy	5	6,185	599	
Holstein	Netherlands	Dairy	6	8,587	995	
Jersey	Jersey	Dairy	8	12,547	78	
Wagyu	Japan	Beef, Draught				10
Brahman	USA	Beef	10	13,006	99	8
**Total**					**6,373**	**58**

^1^Brahman are indicine cattle developed in the US as a composite of Nelore, Gir, Guzerat and Indu Brazil cattle originating in India but imported primarily from Brazil. The remaining breeds are taurine.

^2^Number of contiguous loci spanning at least 200 kb and with a minor allele frequency ≤ 0.01 required to declare a selective sweep region in each breed.

## Methods

### Samples, design, and genotyping

We utilized two data sets comprising SNPs scored in animals that were registered by their respective breed associations and we sampled across male lineages to ensure that the animals were not closely related and that they represented the diversity within each breed. The first data set comprised 6,373 full-blood animals from 13 taurine breeds (*Bos taurus taurus*) including Angus, Braunvieh, Charolais, Hanwoo, Hereford, Limousin, Salers, Shorthorn, Simmental, Brown Swiss, Finnish Ayrshire, Holstein, and Jersey, and the indicine (*Bos taurus indicus*) Brahman breed (Table 1). Genotypes scored in these animals were generated using the Illumina (San Diego, CA) BovineSNP50 BeadChip which assayed 54,001 loci with a median intermarker interval of 37 kb [29,30]. The second data set comprised 58 animals from the Angus, Hanwoo, Simmental, Wagyu and Brahman breeds (Table 1) which were genotyped with a prescreening assay comprising 2,787,037 SNPs with a median intermarker interval of 975 bp that was used by Affymetrix (Santa Clara, CA) in the design of the Axiom Genome-Wide BOS 1 assay [31]. The Angus, Hanwoo, Simmental and Brahman animals genotyped with the AFFXB1P assay were full-bloods except for the Simmentals which were registered purebreds, and not all of these animals were included in the BovineSNP50 data set. Thus the animal samples are partially independent and decreased from 4.4 to 126.8 fold in size between the assays, but the assay resolution, measured as SNP density, increased ~50 fold between the assays.

The sampled breeds were chosen based on their geographical origins, historical uses by human, diverse phylogenetic relationships and because of the availability of at least 40 BovineSNP50 genotyped full-blood individuals. Each BovineSNP50 genotyped individual was registered with its respective breed association and was proven by pedigree-analysis to be full-blood, since some associations (e.g., Simmental, Limousin) allow the registration of crossbred cattle. This sampling strategy was employed to ensure that there would be minimal effects of recent introgression between the breeds following breed formation.

### SNP filtering

All X-linked loci were removed from the analysis due to the greater number of assembly issues that are associated with this chromosome and also because the studied animals were male resulting in a halving of the number of chromosomes sampled for each breed which leads to a reduction in the precision of allele frequency estimation. The remaining BovineSNP50 genotypes were filtered on call rate ≥85% which left a total of 52,942 SNPs. Within the higher density AFFXB1P data, we required SNPs to have a call rate of ≥85% across all 5 breeds and a minimum call rate of 50% within each of the individual breeds. Following filtering 2,575,339 SNPs remained. These thresholds were based upon an empirical examination of the call rate distributions in the datasets (not shown) to retain the largest number of quality SNP in consideration of the within-breed sample size, which was small for the AFFXB1P genotyped individuals.

### Identification of putative selective sweep regions using BovineSNP50 data

The BovineSNP50 data were analysed by breed to identify putative selective sweeps. Because the number of variable loci differed within each breed primarily due to the breed of origin of SNP discovery in the design of the assay [14,15], we required a breed-specific number (Table 1), and at a minimum 5, contiguous SNPs spanning at least 200 kb based upon UMD3.1 coordinates for which no SNP had a minor allele frequency (MAF) > 0.01 to declare a selective sweep. While, on completion, selective sweeps are characterised by the complete loss of variation within the swept region, we allowed MAF ≤ 0.01 to account for genotyping errors, the possibility of new mutations and assembly errors which may have erroneously assigned a variable marker to a sweep region. Determination of the number of contiguous markers within each breed with MAF ≤ 0.01 to define a sweep region required a trade-off between type I error and the size of the detected sweep region. For example, if 15% of SNPs are monomorphic within a breed (Table 1) [29,30], the probability that N contiguous SNPs are monomorphic is 0.15^N^, assuming independence, and in testing 52,942 SNP on 29 autosomes we would expect to find 0.15^N^ × (52,942 – 29 × (N – 1)) regions in which N contiguous SNPs had fixed alleles. For N = 5 this corresponds to 4.0 false positives per breed but only 0.6 false positives per breed when N = 6. While increasing the number of contiguous monomorphic markers decreases the type I error rate, it simultaneously increases the size of the sweep region that can be detected to, on average, (N – 1) × 37 kb. To allow the identification of moderately sized sweeps, we chose an intermediate balance of these conflicting constraints based on the idea that any sweep identified in two or more breeds would almost certainly be real and likely share a common haplotype, while true sweeps found in only one breed would ultimately be independently validated by other studies. For example, assuming one false positive in each breed, there are 52,942 – 29 × (6 – 1) = 52,797 possible locations for a fixed 6 SNP haplotype in each breed and in the second breed there are 11 positions where the two haplotypes may overlap by at least one SNP (ignoring the centromeres and telomeres where the number is less). Thus the probability that the two false positive fixed haplotypes overlap anywhere in the genome is 52,797 × (1/52,797 × 11/52,797) = 0.0002.

### Identification of putative selective sweep regions using AFFXB1P data

We independently analysed the AFFXB1P data by breed requiring a putative selective sweep region to harbour at least 20 contiguous SNPs spanning at least 100 kb, with no more than 5% of the SNP having a MAF > (2M)^-1^ where M was the number of individuals with genotypes for the SNP within the analysed breed. Among the variable SNPs, we further required that no more than 3 be contiguous. These thresholds were set to allow no more than one individual be heterozygous for a SNP within a selective sweep region in the event of genotyping errors and to allow for new variation to have been created within each region by mutation. These conditions again also allow for the possibility that the SNPs may not have been correctly ordered by the UMD3.1 assembly and that variable contigs may have erroneously been included within scaffolds containing a selective sweep. For regions containing 20 contiguous monomorphic SNPs, we would expect less than 10^-10^ false positives per breed if 15% of the SNPs were monomorphic within the breed. However, estimation of MAF for the loci on this assay was also influenced by the small sample size within each breed.

### Annotation and functional analysis

Annotation of the genes present within all putative selective sweep regions was performed using the UCSC Genome Browser [32] and NCBI Gene database. Genes for which annotations were retrieved included any genes that were fully, or partially, contained within each region. Phenotypes known to be affected by variation in these genes were determined from a search of the literature and were assessed for their likely causality for each sweep. Functional analyses were performed for the sweeps detected within each breed using the functional annotation and clustering tools in the Database for Annotation, Visualization and Integrated Discovery (DAVID) [33].

## Results

### Regions identified as harbouring selective sweeps using BovineSNP50 data

**Figure 1:**
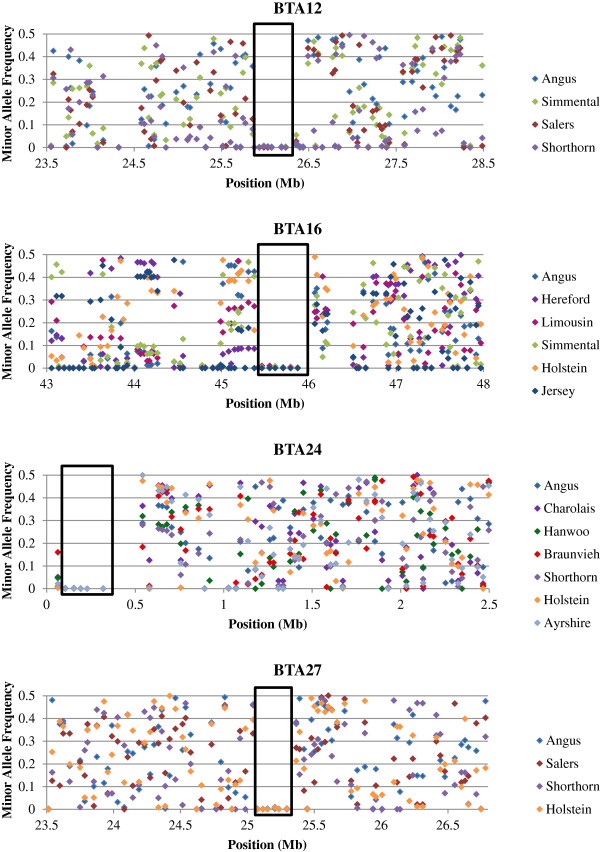
**Selective sweep regions discovered in the analysis of the BovineSNP50 data that were predicted to be common to two or more breeds.** Regions identified as harbouring commonly selected haplotypes are indicated by the near-zero MAF values and are indicated by black boxes.

Twenty eight genomic regions on 15 chromosomes were identified as putatively harbouring selective sweeps (Table 2). Selective sweeps were found in all 14 breeds; however, breed-specific selective sweeps were not identified in every breed. Twenty three predicted sweeps were breed-specific and 5 were shared among two to seven breeds. Four sweep regions were common to at least four breeds (Figure 1). Breed-specific sweeps averaged 336 ± 119 kb and ranged in size from 207 to 702 kb but were not different in size (P < 0.19) to sweeps common to two or more breeds which when calculated separately for each breed (as opposed to the common core identified as the intersection of overlapping sweeps) averaged 441 ± 222 kb and ranged in size from 215 to 866 kb. Common sweeps overlapped but did not have identical boundaries in all breeds, however, the haplotypes found at the core loci in each of these sweeps were identical for each of the breeds in which the sweep was detected. The average number of selective sweeps found in Charolais, Hanwoo, Salers, Brown Swiss and Jersey (all with 78 or fewer animals) was 3.8 while the average number detected in Hereford, Angus and Holstein (all with at least 812 animals) was 4.0 (Table 2) suggesting that variation in sample size did not play a significant role in elevating the false positive rate in the breeds with small sample sizes.

**Table 2:** Putative selective sweep regions identified by analysis of BovineSNP50 genotypes

**Breed**	**BTA**	**UMD3.1 coordinates (bp)**	**# SNP**	**Size (bp)**
Angus^1^	1	1,712,261-2,013,659	11	301,398
Limousin	2	5,974,885-6,344,425	7	369,540
Brown Swiss	2	73,436,684-73,978,469	10	541,785
Hanwoo^1^	3	19,860,064-20,175,158	5	315,094
Hanwoo^1^	3	112,997,892-113,219,287	5	221,395
Brown Swiss	4	61,098,696-61,376,132	9	277,436
Hereford	6	70,655,812-70,865,694	6	209,882
Brown Swiss	6^2^	75,830,633-76,696,893	9	866,260
Salers		75,996,320-76,696,893	8	700,573
Jersey	6	105,390,830-105,730,372	8	339,542
Limousin	7	40,250,259-40,485,825	7	235,566
Jersey	7	45,439,468-45,828,427	10	388,959
Holstein	7	72,908,532-73,126,315	7	217,783
Brown Swiss	11	25,577,969-25,941,999	10	364,030
Jersey	12	1,113,009-1,436,093	8	323,084
Angus^1^, Salers, Shorthorn, Simmental^1^	12	25,878,820-26,236,394	7	357,574
Brahman^1^	12	36,287,533-36,989,957	14	702,424
Holstein	13	15,456,721-15,683,571	6	226,850
Braunvieh	14	28,674,493-28,960,475	5	285,982
Brown Swiss	14	42,739,573-43,140,076	9	400,503
Braunvieh	16	39,714,165-39,898,049	3	389,103
Salers	16	43,200,419-43,618,684	9	418,265
Jersey	16	45,376,614-45,874,144	8	497,530
Angus^1^, Holstein, Limousin, Simmental^1^		45,425,579-45,874,144	7	448,565
Hereford		45,464,423-45,874,144	6	409,721
Hanwoo^1^	16	52,535,473-52,742,523	4	207,050
Hanwoo^1^	18	14,526,709-14,847,049	5	320,340
Limousin	19	37,301,353-37,520,214	6	218,861
Brahman^1^	19	43,376,032-43,835,219	10	459,187
Angus^1^, Holstein, Shorthorn	24	63,576-320,143	6	256,567
Braunvieh, Charolais, Finnish Ayrshire, Hanwoo^1^		105,589-320,143	5	214,554
Angus^1^, Holstein, Salers, Shorthorn	27	25,075,449-25,295,935	6	220,486

^1^These breeds were also genotyped with the AFFXB1P assay and allow the potential validation of these putative selective sweep regions.

^2^Call rates for some SNP within this region did not exceed the 85% threshold that was applied to the remaining data.

Three of the five selective sweep regions detected in two or more breeds involved both beef and dairy breeds, whereas the 358 kb region on BTA12 is common to only the Angus, Salers, Shorthorn and Simmental beef breeds. None of the five selective sweeps shared by two or more breeds are phylogenetically congruent in the sense that we might have expected the sweep to have arisen in a recent common ancestor [3,34]. While the large selective sweep region on chromosome 6 at ~75.9-76.7 Mb is shared by the closely related Salers and Brown Swiss breeds, Salers and Limousin are sister breeds [2,3] and Limousin does not demonstrate evidence of this sweep. This suggests that the mutations were independently selected within these breeds despite the complex history of inter-crossing that occurred during breed development [2,3]. There were no putative sweeps shared in common between any of the taurine breeds with the indicine Brahman breed. The DAVID functional analysis did not yield any significant functional enrichment of gene ontology terms for these sweep regions suggesting that each of the sweeps were based on functionally independent variants that influenced distinct phenotypes.

### Regions identified as harbouring selective sweeps using AFFXB1P data

A total of 85 putative selective sweep regions spanning from 200 to 846 kb and averaging 321 ± 132 kb were identified on 28 of the 29 bovine autosomes in the five genotyped breeds and, of these regions, 20 were shared in two or more breeds (Tables 3 and 4). These regions harboured from 20 to 477 contiguous SNPs with no more than 5% of the SNPs being variable. Among the selective sweeps identified in two or more breeds, the number of breeds included in this analysis was too small to make inferences about the phylogenetic congruence of shared sweep regions, however, three sweeps were found only in the closely related East Asian Wagyu and Hanwoo breeds. All of the breeds that shared a common selective sweep (Table 4) were fixed for the same core haplotype with two exceptions. The sweep on BTA16 at 45,386,065-45,652,672 and on BTA21 at 1,727,412-2,142,823, both shared by the Angus and Simmental breeds, were fixed for haplotypes that differed at a single SNP. The allele that varied in the haplotypes that were swept to fixation on BTA16 was the 7^th^ of 248 SNPs, whereas on BTA21 the variable allele was at the 22^nd^ of 30 SNPs, indicating a conserved core at both loci. No sweeps were found in common between the four taurine breeds and the indicine Brahman.

**Table 3:** Putative breed-specific selective sweeps identified using the AFFXB1P data

**Breed**	**BTA**	**UMD3.1 coordinates (bp)**	**Size (bp)**	**# Total SNP**	**# Fixed SNP**	**# Variable SNP**
Angus	1	1,673,108-2,024,737	351,629	235	231	4
	1	111,659,758-111,900,241	240,483	231	221	10
	3	28,196,188-28,405,853	209,665	207	199	8
	6	6,308,164-6,696,861	388,697	74	72	2
	8	70,780,263-71,227,757	447,494	24	24	0
	8	73,538,014-73,760,303	222,289	288	282	6
	11	99,717,097-99,929,710	212,613	63	60	3
	14	3,550,689-3,885,375	334,686	21	21	0
	14	24,631,146-25,173,007	541,861	387	376	11
	18	200,903-406,270	205,367	117	114	3
	20	70,827,025-71,040,113	213,088	30	29	1
	21	1,186,567-1,489,860	303,293	26	25	1
	21	3,091,822-3,417,143	325,321	308	301	7
	25	39,262,249-39,468,067	205,818	21	20	1
	27	36,633,324-36,896,534	263,210	135	130	5
	29	49,557,166-49,922,377	365,211	70	69	1
Brahman	5	48,679,627-48,903,409	223,782	249	237	12
	10	24,519,718-24,794,435	274,717	20	20	0
	22	10,701,509-10,963,520	262,011	88	86	2
Hanwoo	9	1,384,189-1,586,988	202,799	214	211	3
	22	60,772,158-61,015,491	243,333	24	23	1
Simmental	1	83,467,837-83,685,705	217,868	124	118	6
	2	36,576,839-36,828,533	251,694	216	206	10
	2	119,873,146-120,084,764	211,618	158	151	7
	2	121,398,894-121,698,563	299,669	196	188	8
	5	68,631,784-68,857,802	226,018	276	267	9
	7	21,101,050-21,348,345	247,295	65	63	2
	7	52,426,108-52,673,522	247,414	40	38	2
	8	70,246,437-70,540,424	293,987	163	158	5
	10	59,147,189-59,382,774	235,585	190	182	8
	10	72,811,087-73,041,092	230,005	217	210	7
	13	12,236,039-12,524,368	288,329	382	369	13
	13	66,468,866-66,708,674	239,808	153	146	7
	15	82,007,368-82,208,624	201,256	32	32	0
	16	42,555,966-42,788,613	232,647	46	44	2
	16	43,767,572-44,053,904	286,332	186	181	5
	17	13,291,922-13,492,010	200,088	141	136	5
	19	34,588,859-35,435,279	846,420	59	57	2
	22	2,788,408-3,001,597	213,189	216	214	2
	25	26,433,626-26,744,488	310,862	126	120	6
	25	42,202,212-42,775,697	573,485	21	21	0
	28	30,480,640-30,711,987	231,347	254	243	11
	29	39,045,837-39,396,761	350,924	93	91	2
	29	49,052,295-49,273,683	221,388	21	20	1
Wagyu	1	197,944-574,931	376,987	321	313	8
	1	12,544,028-12,840,164	296,136	335	325	10
	2	71,299,739-71,508,079	208,340	177	169	8
	2	136,434,039-136,676,537	242,498	22	21	1
	3	12,027,611-12,227,923	200,312	65	64	1
	3	113,841,208-114,151,444	310,236	189	181	8
	3	117,817,862-118,017,993	200,131	114	110	4
	4	117,846,325-118,095,397	249,072	21	20	1
	4	119,437,108-119,832,270	395,162	26	25	1
	11	33,020,266-33,264,604	244,338	307	293	14
	11	59,513,020-59,783,144	270,124	282	269	13
	11	103,333,722-103,590,033	256,311	33	32	1
	11	105,596,531-105,828,115	231,584	23	22	1
	12	23,283-377,340	354,057	137	137	0
	13	15,493,906-15,739,251	245,345	305	301	4
	13	58,016,490-58,335,951	319,461	114	110	4
	16	51,275,719-51,519,519	243,800	39	38	1
	19	53,829,568-54,065,288	235,720	25	24	1
	22	49,088,027-49,456,146	368,119	102	98	4
	24	36,584,702-36,795,576	210,874	224	218	6
	27	17,911,399-18,114,646	203,247	304	289	15

**Table 4:** Putative selective sweep regions detected in at least two breeds using AFFXB1P data

**Breeds**	**BTA**	**UMD3.1 coordinates (bp)**	**Size (bp)**	**# Total SNP**	**# Fixed SNP**	**# Variable SNP**
Angus	6	5,639,799-5,993,214	353,415	95	92	3
Simmental, Wagyu, Hanwoo		5,639,799-5,993,214	353,415	95	94	1
Angus, Simmental, Wagyu	6	106,844,116-107,308,280	464,164	21	20	1
Simmental	7	4,344,221-4,677,474	333,253	22	21	1
Hanwoo		4,443,937-4,838,781	394,844	90	88	2
Angus	7	51,000,141-51,430,242	430,101	406	391	15
Simmental		51,144,109-51,788,442	644,333	477	469	8
Wagyu	14	2,098,182-2,588,143	489,961	29	29	0
Hanwoo		2,097,493-2,603,935	506,442	31	30	1
Angus	16	44,463,682-44,881,229	417,547	213	203	10
Simmental		44,693,447-45,207,060	513,613	162	158	4
Angus	16	45,386,065-45,652,672	266,607	248	238	10
Simmental		45,386,065-45,677,279	291,214	286	280	6
Simmental	16	49,400,423-49,679,673	279,250	22	21	1
Wagyu		49,400,423-49,661,831	261,408	20	20	0
Simmental	16	52,629,624-52,857,759	228,135	30	30	0
Wagyu		52,629,624-52,857,759	228,135	30	29	1
Wagyu	17	69,501,174-69,703,717	202,543	116	112	4
Wagyu		69,953,291-70,183,461	230,170	145	140	5
Hanwoo		69,705,850-70,283,199	577,349	421	402	19
Simmental	17	73,619,837-73,975,539	355,702	24	24	0
Wagyu		73,761,834-74,325,363	563,529	57	55	2
Angus	18	14,725,181-14,973,411	248,230	107	102	5
Simmental		14,725,181-14,973,411	248,230	107	104	3
Angus	18	53,342,619-53,596,733	254,114	53	51	2
Simmental		53,342,619-53,573,919	231,300	46	44	2
Angus	19	57,106,999-57,377,040	270,041	26	25	1
Simmental		57,065,941-57,377,040	311,099	28	28	0
Angus	20	71,679,859-72,012,001	332,142	37	36	1
Simmental		71,780,338-72,012,001	231,663	29	28	1
Angus	21	2,134-742,281	740,147	422	408	14
Simmental		2,134-326,342	324,208	187	179	8
Angus, Simmental	21	1,727,412-2,142,823	415,411	30	29	1
Angus	21	70,948,810-71,284,393	335,583	23	22	1
Hanwoo		71,112,766-71,575,370	462,604	32	31	1
Angus	26	21,432-718,976	697,544	242	234	8
Simmental		21,432-723,266	701,834	250	244	6
Wagyu		21,432-225,284	203,852	30	29	1
Wagyu		249,534-454,031	204,497	81	77	4
Hanwoo		21,432-454,031	432,599	121	119	2
Simmental	29	1,156-558,130	556,974	155	153	2
Wagyu		105,179-536,644	431,465	122	120	2
Hanwoo		315,440-558,130	242,690	90	86	4

Since only a subset of the 14 breeds genotyped with the BovineSNP50 assay were also assayed with the AFFXB1P assay, we had the potential to validate only 11 of the putative selective sweep regions identified in Table 2 and five regions were confirmed (Table 5). Only two of the regions were confirmed in the same breeds that led to their identification using the BovineSNP50 assay and for two of the remaining regions, discovery occurred in Hanwoo and confirmation occurred in the phylogenetically similar Wagyu breed [2,3]. However, the region on BTA13 was identified in Holstein using the BovineSNP50 data but was independently validated in Wagyu by the AFFXB1P data.

**Table 5:** Genomic regions predicted to harbour selective sweeps using BovineSNP50 data and validated by AFFXB1P data

**BTA**	**Breed**	**UMD3.1 coordinates (bp)**	**# Fixed SNP**	**# Variable SNP**	**Size (bp)**	**Annotations**
1	Angus	1,673,108-2,024,737	313	8	351,629	Horn-polled [6]
13	Wagyu	15,493,906-15,739,251	301	4	245,345	*DGKZ*
16	Angus	45,386,065-45,652,672	238	10	266,607	*ENO1*, *RERE*
	Simmental	45,386,065-45,677,279	280	6	291,214	
16	Simmental	52,629,624-52,857,759	30	0	228,135	*C16H1orf159, RNF223, F1N376, AGRN, ISG15, HES4, C16H1orf170, PLEKHN1, KLHL17, NOC2L, SAMD11*
	Wagyu	52,629,624-52,857,759	29	1	228,135	
18	Angus	14,725,181-14,973,411	102	5	248,230	*TCF25*, *SPIRE2*, *MC1R*, *TUBB3*, *MIR220D*, *DEF8*, *CENPBD1*, *LOC532875*, *DBNDD1*, *GAS8*, *LOC100296324*, *LOC100336472*, *SHCBP1*
	Simmental	14,725,181-14,973,411	104	3	248,230	

### Annotation and causal candidates underlying selective sweep regions

The putative selective sweep regions were found to harbour annotated bovine protein coding regions or human orthologues, conserved sequences likely to be regulatory elements, and pseudogenes. However, relatively few regions yielded genes likely to be selection candidates based upon identifiable phenotypes (Table 6). Five regions were associated with breed hallmarks such as coat colour and pattern or morphological characteristics. Several regions harboured olfactory receptor-like variants, or genes associated with neurological development or behavioural disorders as well as embryo patterning, survival, and development.

**Table 6:** Potential causal genes underlying selective sweep regions and their associated function or phenotype

**SNP50 discovery breed(s)**	**AFFXB1P discovery breed(s)**	**BTA**	**Sweep position (Mb)**	**Potential causative gene(s)**	Gene or region type^*^	**Associated function or phenotype**
**SNP50 Specific Sweep Regions**						
Hereford	---	6	70.65-70.87	*spotted*^1^	---^1^	white points on face, underline, feet & tail in cattle [8]
Angus, Salers, Shorthorn, Simmental	---	12	25.87-26.23	*NBEA*	PC	autism spectrum disorder in human [35,36]
				*MAB21L1*	PC	psychiatric disorders in human [37]
Angus, Hanwoo, Hereford, Holstein, Limousin, Simmental	---	24	0.06-0.32	*LOC10013911*	PS	olfactory receptor-like variants
				*LOC783097*	PS	olfactory receptor-like variants
				*LOC100337172*	PS	olfactory receptor-like variants
**Co-Discovered Regions (SNP50 and AFFXB1P)**						
Angus	Angus	1	1.67-2.02	*POLL*^*2*^	---^2^	presence or absence of horns in cattle [5,6,38]
Holstein	Wagyu	13	15.49-15.74	*DGKZ*	PC	leptin signaling – linked to human obesity [39]
Angus, Hereford, Jersey, Holstein, Limousin, Simmental	Angus, Simmental	16	45.39-45.65	*RERE*	PC	embryogenesis and embryonic survival [40-42]
Hanwoo	Simmental, Wagyu	16	52.63-52.86	*AGRN*	PC	cell adhesion between embryo & maternal tissues [43]
				*HES4*	PC	oscillation and repression of embryogenesis [44]
				*ISG15*	PC	acknowledgement & maintenance of pregnancy [45,46]
Hanwoo	Angus, Simmental	18	14.72-14.97	*MC1R*	PC	black coat colour in cattle [7]
**AFFXB1P Specific Sweep Regions**						
---	Simmental	1	83.46-83.68	*HTR3E*	PC	serotonin receptors – social cognition (tameness)
				*HTR3C*	PC	in foxes and dogs [47-50]
---	Angus	1	111.66-111.90	*LEKR1 CCNL1*	PC	reduced birth weight in humans [51]
---	Angus	3	28.19-28.40	*TSPAN2*	PC	oligodendrocyte signaling & maturation [52,53]
---	Brahman	5	48.68-48.90	*MSRB3*	PC	ear floppiness and morphology in dogs [54,55]
---	Angus, Hanwoo, Simmental, Wagyu	6	5.64-5.99	*MAD2L1*	PC	proper onset of anaphase during cell division
---	Angus, Simmental, Wagyu	6	106.84-107.30	*RGS12*	PC	positional candidate for ovulation rate in swine [56]
---	Wagyu	11	33.02-33.26	*NRXN1*	PC	synaptic function – linked to autism spectrum disorders [57,58]
---	Simmental	13	66.46-66.70	*SAMHD1*	PC	innate immune response [59]
---	Angus	14	24.63-25.17	*PLAG1*	PC	variation in human height [60] and cattle stature [61]
---	Angus, Simmental	16	44.69-44.88	*PIK3CD*	PC	immune response
				*SPSB1*	PC	immune regulation [62]
---	Simmental, Wagyu	17	73.76-73.97	*VpreB*	PC	B cell development
				*PRODH*	PC	schizophrenia [63]
---	Angus, Simmental	20	71.67-72.01	*IgCgamma*	PC	immune response
				*PDCD6*	PC	T cell receptor-induced apoptosis
---	Angus, Hanwoo	21	70.11-71.28	*AHNAK2*	PC	skeletal muscle fiber organization [64]
				*IGHE*	other	immune response
				*CRIP1*	PC	immune regulation [65,66]
				*CRIP2*	PC	immune regulation [65,66]
---	Brahman	22	10.70-10.96	*TRANK1*	PC	bipolar disorder in humans [67]
				*DCLK3*	PC	brain development [68]
				*GOLGA4*	PC	golgi identity/structure [69]
				*ITGA9*	PC	gamete interaction & reduced fertilization success [70]
---	Wagyu	22	49.08-49.45	*TLR9*	PC	innate immunity
---	Simmental	25	26.43-26.74	HSA16^3^	HO	autism spectrum disorders [71-73]
---	Angus, Hanwoo Simmental, Wagyu	26	0.21-0.45	*OR5D18*	PC	olfactory receptor [74]
				various loci^4^	PS	olfactory receptor-like variants
---	Wagyu	27	17.91-18.11	*CBX1*	PC	epigenetic reprogramming of embryo/gene expression [75-77]
---	Simmental	28	30.48-30.71	*ADK*	PC	physiological state – blood flow, neurotransmission in brain [78]
---	Hanwoo, Simmental, Wagyu	29	0.32-0.56	*ANKRD26P3*	PS	ncRNA regulatory activity
				*CCDC144C*	PS	ncRNA regulatory activity

*Gene and region types are defined as: *PC* protein coding, *PS* pseudogene, *HO* human orthologue, other = known type, unspecific.

^1^The causal mutation has not yet been identified but the locus has been fine mapped to this region, see reference.

^2^The *POLL* locus in cattle has been identified but is characterised by allelic heterogeneity among breeds.

^3^This region is orthologous to human chromosome 16 (HSA16).

^4^There are 13 loci identified as olfactory receptor-like variants which have been determined to be pseudogenes.

## Discussion

We utilised two genotyping assays to identify putative selective sweep regions within the bovine genome. The BovineSNP50 assay was employed because we have genotyped a large number of registered animals from several breeds with this assay; however, we recognise that the assay is not ideal for this purpose due to the ascertainment of common SNPs in its design. Since the *Bos taurus taurus* breeds in Table 1, and Angus and Holstein in particular, were used for SNP discovery and SNPs with high minor allele frequencies in these breeds were preferentially included during the design of the assay [14,15] it is clearly unsuited to the identification of selective sweep regions that might be common among breeds. However, SNPs were included in the design of the BovineSNP50 assay if they were found to be variable in several, but not necessarily all of these breeds. Therefore, the assay theoretically possesses the ability to identify selective sweeps that are specific to individual breeds or to a small number of breeds. However, rather than characterising sweeps that occurred during the domestication of cattle and that should therefore be common, e.g., among European taurine breeds that descended from cattle that were domesticated in the Fertile Crescent, these sweeps are much more likely to have occurred during the formation of breeds and will reflect selection to fix phenotypes such as coat colour or the absence of horns within specific breeds.

A second limitation of this assay is that of calibration relative to the size of the sweep regions. While strong sweeps in numerically small populations are expected to result in the fixation of large haplotypes, weak selection in numerically large populations will result in the fixation of only a small core haplotype which may not be detected using this assay. Thus, historic variation in the census population size among breeds may have resulted in variation in the size of the fixed haplotype and our inability to detect small haplotypes. By requiring N contiguous loci to each have a minor allele frequency (MAF) < α, for small α, we must choose N to be sufficiently large that it would be highly unlikely to observe N contiguous loci all with a MAF < α due to chance alone and yet sufficiently small that the targeted sweeps are not smaller than 37 × (N-1) kb, where 37 kb represents the median intermarker interval on the BovineSNP50 assay. The design of the BovineSNP50 assay also led to lower average MAF and larger numbers of monomorphic loci in breeds such as Brahman, that are phylogenetically distant from the SNP discovery breeds [14]. To adjust for this bias, we defined N separately for each breed (Table 1) requiring larger N for breeds with larger numbers of monomorphic and low MAF loci. The definition of α > 0 is also important to this discussion since in the detection of sweeps we must allow for old sweeps in which *de novo* mutations may have begun to accumulate on the fixed haplotype, genotyping errors which are locus specific but average about 0.5% for this assay, and the incorrect ordering of loci by the UMD3.1 sequence assembly. However, errors in the assembly are vastly more likely to cause false negative than false positive sweeps by incorrectly introducing a variable locus into a region of dramatically reduced variability within the genome.

We also employed the AFFXB1P assay which contained almost 2.8 million putative SNPs that were screened for variability in a small number of animals from several breeds prior to the design of the commercial BOS 1 Axiom assay. While we had many fewer animals genotyped with this assay which influenced the estimation of MAF, the AFFXB1P assay had over 50× the number of SNPs present on the BovineSNP50 assay which offered considerably greater power for identifying small sweeps and the application of this assay also suffers less from ascertainment bias. While loci that have been fixed in all domesticated cattle relative to their auroch forbears will still not appear on this assay due to the requirement that the putative SNP must have been predicted to have been variable in the sequence data for at least one breed, there was much less selection for SNPs with high MAF in numerous breeds in the design of this assay relative to the BovineSNP50. Consequently, we expected this assay to identify putative sweep regions that could not be identified by the application of the BovineSNP50 assay and to more precisely define the boundaries of sweeps that were detected by the BovineSNP50 assay and validated by the AFFXB1P assay.

One of the main focuses of this study was to identify selective sweep regions for which a single haplotype was found in all breeds at the core region defined as the intersection of the overlapping sweep regions found in each of the breeds. The existence of these shared core regions dramatically reduces the potential for false positive discoveries since the likelihood of extended identical haplotypes being found in multiple breeds due to chance alone is close to nil. We identified a handful of regions in each dataset that met these criteria. The putative sweep shared among six breeds on BTA24 contains four pseudogenes, of which three are olfactory receptor-like. Whether any of these pseudogenes are expressed is unclear; however, olfactory receptor loci were detected as being recently duplicated within the bovine genome [79] suggesting that they may also be under strong selection for newly evolving functions. The common sweep region on BTA12 contains neurobeachin (*NBEA*) and mab21-like 1 (*MAB21L1*) which have been implicated in human autism and psychiatric disorders, respectively [35-37]. Since these phenotypes represent extreme behaviours, it is intriguing to speculate that mutations in these genes may also predispose cattle to increased docility and more favorable temperaments when handled by humans.

Of the regions identified in both datasets, a selective sweep on BTA13 was detected in Holstein using the BovineSNP50 data and was also discovered in Wagyu using the AFFXB1P data. The region from 15.49-15.74 Mb contains diacylglycerol kinase zeta (*DGKZ*) and several bovine ESTs. *DGKZ* has been implicated as a member of the downstream leptin signaling pathway and reduced expression or activity within the hypothalamus has been associated with obesity [39]. The Holstein and Wagyu breeds are phylogenetically distant, however, Wagyu are believed to have been influenced by several European taurine breeds, including Holstein, during the late 1800s and both breeds are known for their ability to store intramuscular fat without accumulating excessive subcutaneous fat [80]. The 351 kb selective sweep region from 1.67-2.02 Mb on BTA1 found in Angus using the BovineSNP50 and validated in Angus using the AFFXB1P data contains a fixed 321 marker haplotype which harbours the *POLL* locus [6] a hallmark of the breed which contains only polled animals.

A region on BTA18 from 14.72-14.97 Mb was detected to harbour a selective sweep in Hanwoo cattle using the BovineSNP50 data and rediscovered in Angus and Simmental using the AFFXB1P data. This 248 kb region contains several annotated genes (Table 5), but importantly harbours melanocortin 1 receptor (*MC1R*) in which mutations lead to the black coat colour in cattle [7]. American Angus have been strongly selected for black coat colour and almost all animals registered by the American Angus Association are now homozygous black confirming that the basis for this selective sweep in Angus was for the black coat colour allele. The American Simmental Association registers animals that have been upgraded to purebred status (7/8ths Simmental) and many breeders have graded up to purebred animals from Angus crossbreds to capitalize on the premium that carcasses from black coated cattle can achieve if they qualify for Angus branded products. While all of the Simmentals genotyped with the BovineSNP50 assay were full-blood, the 6 Simmentals genotyped with the AFFXB1P assay were all purebred with one animal being identified as homozygous black and another four with BLACK incorporated into their registered names. Since the economic advantage is maximized for bulls that produce 100% black calves, we speculate that at least one-half, and quite possibly all, of the chromosomal segments found in this region in these Simmentals actually originated in Angus. The fact that the sweep in Angus was not found using the BovineSNP50 data suggests a resolution issue with the requirement that at least 6 contiguous loci spanning at least 200 kb be fixed in order to declare a sweep. On the other hand, the fact that a sweep was detected in the AFFXB1P data for Simmental that was not detected using the BovineSNP50 data suggests a sampling issue, since the animals genotyped with the BovineSNP50 were all full-blood whereas the 6 Simmentals genotyped with the AFFXB1P were all purebred and selected to have black coat color. The result for Hanwoo is more interesting since the sweep was declared in Hanwoo using the BovineSNP50 data for 48 full-blood individuals but was not confirmed in the 11 full-blood individuals genotyped with the AFFXB1P data. This suggests that either the region is not correctly assembled, or that an ancient breed foundation event may have occurred in which the yellow allele was fixed in this breed, but that sufficient mutation events have occurred on this *MC1R* haplotype to cause it to fail to be detected as a sweep using the ultra-high-density data. Finally, of particular interest is the fact that no sweep was identified in this genomic region in Wagyu cattle suggesting that black coat colour in Angus and Wagyu cattle may not be allelic. Recently, a mutation within beta-defensin 103 (*CBD103*) has been shown to cause black coat colour in dogs [81]. The cattle ortholog of *CBD103* maps to 4.89 Mb on BTA27 centromeric of the sweep that was detected in Wagyu cattle (Table 3).

**Figure 2:**
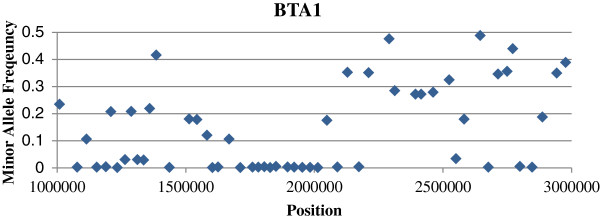
**Selective sweep surrounding the *POLL *locus in Angus cattle.** The selective sweep region on BTA1 in Angus is from ~1.7 Mb to 2.0 Mb and contains the *POLL* locus. The locus is contained within an extended region of reduced diversity relative to the up- and down-stream SNPs.

After analysing regions identified in multiple breeds, we sought to identify any potential phenotypes under selection within breed-specific regions. Within the lower density BovineSNP50 data, we identified a sweep region towards the centromere of BTA1 harbouring 11 contiguous monomorphic SNPs and spanning 301 kb in Angus (Figure 2). This region contains the *POLL* locus [6] for which this breed has been strongly selected for homozygosity of the *POLL* allele [38]. Hereford cattle are also homozygous for the dominant *spotted* allele at the *spotted* locus which is a candidate for the 210 kb sweep region at 70.65-70.87 Mb on BTA6 [8]. The *spotted* locus affects the white points on the face, underline, feet and tail which are a characteristic of the Hereford breed. These breed-specific sweeps are clearly examples of strong selection on loci which underlie phenotypes that are hallmarks of certain breeds and where the underlying causal mutation is known or has been mapped to a chromosomal location.

Among other selective sweep regions identified using the BovineSNP50 data, several contain no annotated genes. Either this reflects the incomplete annotation of the bovine genome, or the fact that the selected functional mutation within each of these regions is not located within a protein coding gene. Examining each of these regions for the alignment of human, pig and sheep mRNA orthologs failed to identify any genes. Recent work has identified ncRNAs which regulate the expression of nearby genes [82] and may help identify candidates for the mutations in these regions that were subjected to selection. A region on BTA29 found in Hanwoo, Simmental, and Wagyu breeds was identified as containing ncRNA regulatory activity (Table 4).

Among the other detected breed-specific sweep regions, there were several regions linked to known phenotype to genotype associations. A region on BTA14 specific to Angus cattle harbours several genes including *PLAG1* which has been associated with variation in human height [60], the stature of cattle [61] and with vertebrae number and back elongation in swine [21]. Angus has been strongly selected for growth and frame size during the last 30 years [83] perhaps creating this selective sweep. The telomeric sweep region on BTA1 found in Angus contains several genes, including *LEKR1*and *CCNL1* in which mutations have been associated with reduced birth weight in humans [51]. Angus cattle have recently been selected to reduce birth weights to ease dystocia [34]. Another region of particular interest, and perhaps the most interesting identified within the indicine sample, was detected in Brahman and located at 48.68-48.90 Mb on BTA5 and contains the methionine sulfoxide reductase B3 (*MSRB3*) gene which has previously been identified as a candidate for a QTL affecting ear floppiness and morphology in dogs [54,55]. Brahman cattle were developed in the US as a cross between the *Bos taurus indicus* breeds Guzerat, Nelore, Gir and Indu Brazil imported primarily from Brazil but all originating in India and the *Bos taurus taurus* Shorthorn and Hereford breeds [84]. There is considerable variation among these breeds for ear length and morphology with Indu Brazil animals having particularly large, pendulous ears. Thus, the sweep in this region may reflect strong recent selection by breeders to establish a specific Brahman ear morphology type. Key fitness traits such as behaviour and reproduction are postulated to underlie the sweeps detected in multiple regions on chromosomes 6, 11, 12, 22, 25, and 28 (Table 6). The link between genes involved in psychiatric disorders poses a potential link to selection for improved temperament in cattle. Mutations in these behavior-associated genes may confer improved temperament when cattle are handled by humans and these would have been strongly selected following breed formation to develop more manageable animals. The presence of sweeps in regions harbouring genes associated with reproductive processes may be a result of the selection for mutations which enhance reproductive rate or that result in improved calving ease.

An interesting observation relative to the identified breed-specific and common selective sweeps is the enrichment of genes related to immune function and response which have clearly been important to the adaptive evolution of the species [27]. Strong selection for immune function may have occurred following the exposure of animals to new pathogens during changes in management at domestication and breed formation, and after their introduction to North America where these animals were sampled. Adaptive evolution of the immune system has been seen in many species such as *Drosophila*[85,86] and humans [10], as well as plants [87].

The abundance of olfactory receptor (OR) genes and pseudogenes within sweep regions is intriguing and suggests that olfactory loci play a major role in the domesticability of species. Olfactory receptor genes have previously been found to have been under selection in cattle [12,25] and more recently in swine [88] and it has been hypothesized that pigs rely intensely on their sense of smell for scavenging. Alterations in the need for wild animals to search for food following their domestication may result in a relaxation of the need for purifying selection acting on these genes allowing them to freely evolve to gain new functions in odorant and tastant detection. In tetrapods, anywhere from 20 to 50% of OR loci exist as pseudogenes [89] and while it is not clear if these genes were ever functional, the acquisition of trichromatic vision has been postulated as facilitating the loss of OR genes [90]. On the other hand, cattle are dichromatic and yet still possess significant numbers of OR pseudogenes [89] some of which were found to have potentially been under strong selection in this study. If these pseudogenes lack functionality, we might have expected them to have been deleted from the genome or to have been significantly disrupted by mutation. However, as many as 67% of OR pseudogenes are expressed in human olfactory epithelium [91] suggesting that similar percentages of bovine OR pseudogenes are also expressed and that many of these loci are functional and rapidly evolving in copy number [79].

We found evidence for selective sweeps in genomic regions that were detected to have diverged between breeds using integrated haplotype scores (iHS) and F_ST_ statistics in the Bovine HapMap project [12]. Putative sweep regions overlapped on chromosomes 2, 11, 12, and 14 detected either by an extreme F_ST_ or iHS value. We found evidence for only one selective sweep region for which the Bovine HapMap project [12] identified divergence between breeds using F_ST_ statistics. Using the BovineSNP50 data, we found a putative sweep in Angus, Salers, Shorthorn and Simmental cattle on BTA12 in a region harbouring *NBEA* that was found to have differentiated among taurine breeds. The fact that only one such region was detected is not surprising since regions that have been strongly selected for a derived allele in one breed are likely to be selectively neutral in other breeds which do not possess this allele, leading to small differences in flanking SNP allele frequencies and modest F_ST_ statistics. Large F_ST_ statistics imply divergent selection for alternate alleles within different breeds suggesting that there may be several mutations in *NBEA* that have been strongly historically selected in some breeds and that are currently under selection in others. None of the putative sweep regions detected in the Holsteins were concordant with regions detected to be responding to recent selection in Israeli Holsteins [92]. Rather than reflecting differences in the origin of the founders of the US and Israeli Holstein populations [93], this more likely reflects the fact that our study focused on the identification of loci where selection had driven the desirable allele to fixation, whereas the Israeli study focused on the identification of loci currently responding to strong selection.

Two breed-specific sweep regions in Limousin on BTA2 at 5.97 Mb and BTA7 at 40.25 Mb (UMD3.1 positions) were also identified in a scan of West African cattle for loci underlying adaptive divergence between populations [27]. The region on BTA2 contains *HIBCH*, *MGC128040*, *MSTN*, *PMS1*, *ORMDL1* and *ASNSD1*, of which *MSTN* is almost certainly the locus selected in Limousin due to its effects on muscling. This locus is unlikely to have been divergently selected in the West African populations. Likewise, the region on BTA7 contains several genes (*PRR7*, *DBN1*, *PDLIM7*, *DDX41*, *FAM193B*, *TMED9* and *B4GALT7*) and it is likely that different loci were under selection in U.S. Limousin and West African cattle. In comparison to a study of regions of differentiation among the genomes of three French dairy breeds [28], the coat colour (*MC1R*) locus on BTA18 and the region on BTA14 at 24.63 Mb (UMD3.1 position) harbouring *PLAG1* which is associated with cattle stature [61] were identified in both studies. Also of interest is the fact that platelet-derived growth factor alpha polypeptide (*PDGFA*) was identified as a potential candidate gene underlying the selective sweep at 42.2-42.8 Mb on BTA25 in Simmental, whereas, the receptor for this growth factor (*PDGFRA*) was identified as differentiated among the French dairy breeds. Our findings also demonstrate concordance with a study in dairy cattle where 1,600 out of 34,851 (4.59%) SNPs showed signatures of on-going selection via iHS test statistics [94]. However, the fundamental difference between these studies is that we sought to find loci which had completed selective sweeps whereas this dairy study [94] sought to identify loci that were currently responding to selection and it is not obvious that there are significant numbers of loci for which a sweep has been completed in some breeds but that selection is on-going in others.

No putative selective sweep regions were found in common between Brahman and any of the *Bos taurus taurus* breeds which likely reflects the recent admixture that occurred in the formation of the Brahman and the fact that the breed does not share a common phenotype such as coat colour with any of the taurine breeds. Furthermore, indicine cattle are more commonly found in the southern tier of the US where they are exposed to higher temperatures and humidities and lower pasture qualities and availability than are taurine cattle which are more frequently found in the northern US. Consequently, we would not expect these breeds to have been subjected to selection for common morphological or adaptive phenotypes. Additionally, no common sweeps were detected between the cattle sub-species possibly due to the more severe ascertainment bias on MAF for BovineSNP50 loci in Brahman cattle. While SNP discovery was performed in Brahman for the development of the AFFXB1P assay, the number of indicine breeds sequenced for SNP discovery was small relative to the number of sequenced taurine breeds leading to a bias towards SNPs common in taurine cattle being included on the assay. However, the density of SNPs on this assay is so great (~1 SNP/kb) that we did not expect the reliability of sweep regions identified in Brahman to be significantly less than those identified in taurine breeds. Our identification of a putative sweep region harbouring a previously identified QTL for ear length and floppiness in Brahman is consistent with the introduction of the undesirable allele from Indu Brazil cattle during breed formation and subsequent strong selection by breeders to remove the allele and fix a shorter ear type within the breed.

High-density assays, such as the BovineSNP50, have previously been shown to be adequate for the identification of runs of homozygosity (ROH) and for estimating inbreeding coefficients within cattle breeds [95]. However, this is not the case for the detection of selective sweeps which typically span smaller regions of the genome than ROH which are frequently due to consanguinity and which may represent as much as 12.39% of the genome [95]. We found high-density (~50,000) SNP data to be generally inadequate for the detection of selective sweeps due to the poor calibration of SNP density relative to the size of the targeted sweep regions. Relaxation of the number of contiguous SNPs with fixed alleles in order to detect smaller sweep regions leads to an elevation of type I error rate. Strong, recent selective sweeps causing the fixation of large haplotypes may be identified using high-density SNP panels, however, older sweeps which have accumulated new mutations and weak sweeps which have resulted in the fixation of relatively small haplotypes will not be detected.

Several putative selective sweeps identified using the BovineSNP50 data failed to be validated using the AFFXB1P data. While many fewer animals representing each breed were genotyped with the AFFXB1P assay, among the 50× additional SNPs within each such region, we found that at least 5% of the SNP had a MAF > (2M)^-1^ where M is the number of genotyped animals. We have previously found that the genotyping error rate of loci on the Affymetrix Axiom BOS 1 assay is very similar to that of the Illumina BovineSNP50 assay (~0.5%, data not shown) and thus, we do not expect genotyping errors to explain this result, although it is certainly a possibility. It appears that the phenomenon is either due to errors in the genome assembly or mapping of probes for the AFFXB1P loci, or is simply due to type I errors. As a consequence, the reliability of declaration of a selective sweep is dramatically improved when sweeps are found to be common between breeds, particularly when the breeds are phylogenetically distant. We found several sweep regions that were common to two or more breeds and five sweeps predicted from the BovineSNP50 data were validated by the AFFXB1P data.

Identifying the mutations that underlie these sweep regions will be paramount to more fully understanding the effects of human interaction on the genomes of domesticated cattle. Candidates will soon become available by sequencing the genomes of individuals that are homozygous for identical SNP haplotypes within a sweep region but where some originate from the breeds predicted to have undergone a selective sweep and the others from breeds in which no sweep was detected. However, even after these mutations have been identified, our understanding of the phenotype that was created and selected to complete fixation may still be limited. The functional analysis of genes within the selected regions sheds little light on this since each mutation within these genes may lead to unpredictable phenotypes. Finally, while our sampling of breeds was small, we found little evidence for the sharing of sweeps among phylogenetically closely related breeds. This further supports our conjecture that the design of the BovineSNP50 assay to include common variation makes it primarily suitable for the detection of sweeps that have occurred following breed formation.

## Conclusions

We identified selective sweeps that primarily appear to have occurred following breed formation events. Due to the constraint that SNPs be variable in multiple breeds which was imposed during the design of both of the utilized assays, we did not identify any sweeps that were common to all breeds within the study. There were also no sweep regions predicted to be in common between breeds of taurine and indicine descent probably reflecting the different environmental and demographic forces to which these sub-species have been exposed during breed formation. For several of the detected sweep regions we were able to identify the phenotypes and genes that were subjected to selection, or to propose these based upon the results of previous mapping studies. However, for many of these regions the selected gene and phenotype are unclear. The fact that so many of the detected sweep regions harbour genes associated with behavioural characteristics, immunity, reproductive processes, or embryonic development is probably not remarkable considering the fact that strong selection acts on these fitness traits and that the time required to achieve fixation of variants of modest effect may be considerably longer than the 200 years since breed formation during which strong human selection has acted.

We demonstrate that the resolution and SNP ascertainment bias inherent in the design of the assay used to detect selective sweeps is of paramount importance and that the BovineSNP50 assay is not generally suitable for this purpose due to the high type I error rates that are likely to be encountered. SNP ascertainment bias leads to lower MAF in breeds that are phylogenetically distant from the SNP discovery breeds and an increased rate of monomorphic SNPs within these breeds. As whole genome sequencing becomes less expensive, these problems will likely be ameliorated by sequencing a few distantly related individuals from each breed and this approach may also be used to identify the candidate mutations which underlie each sweep. However, the approach is reliant on the alignment of sequences to a draft Hereford reference assembly [79] which introduces a new set of biases unless *de novo* sequence assemblies can accurately be created for each breed.

The identification of genes and variants underlying historical selective sweeps is of interest from the perspective of understanding how human interaction with cattle has influenced the patterning of variation within the bovine genome. Perhaps of more importance, the discovery of the selected variants will lead to the identification of large effect QTLs and ultimately a better understanding as to the phenotypes which are affected by variation within genes and regulatory elements.

## Data availability

Genotypes are available to scientists interested in non-commercial research upon signing a Materials Transfer Agreement (MTA).

## Competing interests

The authors declare that they have no competing interests.

## Authors’ contributions

JFT, JED and HRR designed the experiment. HRR, RDS and JFT analysed data. SDM, MMR and RDS extracted DNA. MMR and SDM prepared samples for genotyping, SDM ran the Illumina assay, and RDS genotyped samples and managed the genotype database. HRR and JFT wrote the manuscript. All authors read and approved the final manuscript.
